# Impact of Nutrients on the Hoof Health in Cattle

**DOI:** 10.3390/ani10101824

**Published:** 2020-10-07

**Authors:** Lucie Langova, Ivana Novotna, Petra Nemcova, Miroslav Machacek, Zdenek Havlicek, Monika Zemanova, Vladimir Chrast

**Affiliations:** 1 Department of Animal Morphology, Physiology and Genetics, Faculty of AgriSciences, Mendel University in Brno, Zemedelska 1, 613 00 Brno, Czech Republic; xlangov2@mendelu.cz (L.L.); novotna.ivca@post.cz (I.N.); xnemcov6@mendelu.cz (P.N.); xklime12@mendelu.cz (M.Z.); chrast.vl@seznam.cz (V.C.); 2 Department of Animal Protection and Welfare and Veterinary Public Health, University of Veterinary and Pharmaceutical Sciences, Palackeho trida 1, 612 42 Brno, Czech Republic; machacekm@vfu.cz

**Keywords:** dairy cows, lameness, nutrition, vitamins, biotin, minerals, zinc, laminitis

## Abstract

**Simple Summary:**

Nutrition is a significant factor in healthy hoof horn growth. Some minerals, amino acids, and vitamins are involved in the keratinization process, which ensures healthy horn growth and the structural binding of keratin proteins. Laminitis is one of the most important diseases of cattle hooves. Hemorrhage and foot ulcers are considered symptoms of subclinical laminitis. Excess protein in the feed ration can be problematic due to encouraging faster horn growth. The amino acids cysteine and methionine are essential in promoting the structural and functional integrity of the hoof. Currently, various studies focus on the limb health of cattle to obtain information for the prevention or acceleration of the healing of various hoof lesions based on nutritional supplements. Mineral and vitamin (especially biotin) supplementation is associated with a reduction in the incidence of lameness in dairy cows.

**Abstract:**

Lameness is currently one of the most important and economically demanding diseases in cattle. It is manifested in a change in locomotion that is associated with lesions, especially the pelvic limbs. The disease of the hoof is painful, affecting the welfare of dairy cows. Important factors that influence the health of the limbs include nutrition, animal hygiene, stable technology, and genetic and breeding predispositions. Nutrition is one of the basic preventive factors affecting the quality and growth of the hoof horn, and the associated prevalence of hoof disease. The strength and structure of the hoof horn are affected by the composition of the feed ration (amino acids, minerals, vitamins, and toxic substances contaminating the feed ration, or arising in the feed ration as metabolites of fungi).

## 1. Introduction

The quality of hooves has mainly been studied in dairy cows in intensive production systems with a focus on horn health, lameness, and their effect on longevity. The desired hoof quality is defined as normal horn growth that provides enough structural strength, which is the result of the relationship between nutrition, health, and stable conditions [1]. The health of the hooves and the skin adjacent to them are most affected by nutrition and housing technology. Healthy horn growth is only possible if the relevant hoof skin tissue involved is adequately supplied with blood with a balanced content of nutrients and minerals. The hoof provides protection in the distal part of the limb and it is formed by the keratinization of the epithelial layer and modification of the underlying dermis and it is particularly resistant to mechanical and chemical damage. Nutritional disorders, such as acidosis, can lead to excessive lactic acid production, histamine production, and the release of endotoxins, which are all negatively related to hoof health in cattle. The result is bleeding in the horn of the foot, laminitis, and various forms of hoof ulcers [2].

Lameness is currently one of the most critical problems in dairy farming, affecting animal health and welfare [1,3,4,5,6,7,8,9,10,11,12,13,14,15]. Lameness is described as a clinical symptom, which is recognizable by a change in locomotion, usually associated with lesions of the pelvic limbs. More than 90% of diagnosed lesions affect the distal part of the limb [11,13,15,16,17,18,19,20]; however, the resulting lesions are not only a welfare problem, but they also affect production and profit [8,14,15,19,21,22]. Dairy cows that are affected by limb diseases have lower dry matter intake and milk yield [3,4,9,10,11,14,16,22,23,24,25,26,27,28], reduced fertility [3,4,15,16,17,23,24,25,26,27,28,29,30,31,32], and they have an increased risk of culling [3,4,11,14,16,17,18,19,23,24,25,26,27,28,30,31,33].

Lameness of dairy cows is, along with mastitis and reproduction, the third most expensive disease to treat [5,6,7,15,16,20,22,25,26,27,31,32,34,35,36,37,38]. The study by Kaniyamattam et al. [39] reports lameness is second only to mastitis [39]. Economic losses in the affected herds are associated with treatment costs and the decrease in milk production [6,9,14,15,17,18,23,24,27,28,31,33,40,41,42]. Direct costs include treatment, a visit to a veterinarian or trimmer and the time required, the cost of extra time for farmers, and the cost of discarded milk. Indirect costs include, in particular, a reduction in milk production, culling, and an extension of the calving interval [28]. For example, the direct costs of lameness that were caused by digital dermatitis were 121 GBP, interdigital dermatitis 76 GBP, and 152 GBP for ulcers per cow affected; including reduced milk production, the total costs were 240 GBP, 131 GBP, and 425 GBP per cow, respectively [17]. The Burger study [26] suggested that 27% of the costs of production diseases is due to lameness problems.

Limb disease can also occur subclinically and, therefore, may not be directly associated with lameness. However, the impact of hoof disease is practically the same, whether clinical or subclinical [9]. In the clinical form, dairy cows are lame or even unable to stand up due to severe pain in the hoof area. However, lameness often occurs in a subclinical form, meaning that dairy cows can walk, and many are not lame. The subclinical form may affect the level of production, which begins to decline, and it is, therefore, necessary to monitor all possible symptoms for early diagnosis in order to prevent the development to a chronic form of the disease. Decreased milk production often manifests itself before the diagnosis of lameness, which clearly emphasizes the importance of early diagnosis of subclinical lameness [31].

## 2. Prevalence of Lameness

The results of the studies (Table 1) have clearly shown that many herds of dairy cows have problems with lameness [17,43].

The prevalence of lameness is generally estimated at around 25–35% [24] and 25–58% [9]. Rajnbar et al. [6] reported 18.9% (range 5% to 44.5%); in grazed dairy cattle, the prevalence is from 8 to 31% [34]. The study conducted by Dendani-Chadi et al. [36] reported that the prevalence of lame cows was significantly associated with pasture, in non-grazing cows (35.4%), in partially grazing (16.4%), and grazing throughout the year (1.3%). Another study estimated that a total of 75% of cows in Europe are affected by hoof disorders [9]. Sjöström et al. [25] reported results with an overall prevalence of lameness of 18% and in some herds up to 79%, but herds without lame cows were also recorded. Agricultural conditions that may impact lameness vary from country to country and are therefore likely to differ systematically in prevalence. The overall prevalence of lame cows was 18%, with considerable differences between the countries monitored and the individual areas within these countries. However, the prevalence of limb diseases may be much higher in some herds and positively correlates with increasing milk production. The number of breeds in which digital dermatitis and foot rot occur is growing [4]. A study conducted in the United Kingdom of seasonally grazed cows showed that the four most common lesions reported by veterinarians were sole ulcer (15.9%), white line disease (15.5%), digital dermatitis (6.7%), and interdigital phlegmon (6.2%) [46]. The study by Sadiq et al. [13] reported hoof lesions in 87.5% of cows with the highest incidence in cows affected by single lesions (54.2%) and white line disease (61.2%).

## 3. Factors Influencing the Occurrence of Lameness

Lameness is a significant multifactorial disorder [6,7,9,14,15,23,27,30,38,41,46,58,59,60,61,62,63] affecting organic and conventional dairy farms [58,60]. It is mainly at the forefront, due to the increasing prevalence and because no other common dairy disease is associated with such visible symptoms of pain [9,27,60,64], which may be of a long-term nature. Thus, in addition to adverse effects on production, lameness is associated with pain [6,11,22,25,26,31,32,36,40,46,55,60,65,66,67] and anxiety [60]. In this case, the dairy cow cannot exhibit natural behavior [6,40,55,63,68].

Dairy cows are exposed to a variety of environmental influences and related stressors during their productive lives [1,35,63], and healthy hooves are essential in cattle breeding due to their significant impact on functional viability and subsequent efficiency [1]. Most of the factors that lead to the culling of dairy cows are related to health (mastitis, lameness, and reduced fertility) and low production [66,69]. Risk factors that considerably impact limb disease include management [1,9,14,38,41,62,70,71], which plays a significant role in the prevention of lameness, especially nutrition management, and the environment [1,6,8,9,14,22,25,27,30,35,36,41,58,61,62,71], genetics [4,8,14,62,71], and various breeding conditions and technologies [7,22,62]. 

### Nutrition

Nutrition is a significant factor affecting the healthy growth of the hoof horn [4]. The composition of the feed ration significantly affects the growth, development, and health of the hooves [13,26,72,73]. Research has shown that some of the components of the feed ration (minerals, vitamins, amino acids, and fatty acids) play a substantial role in the structural integrity of the hooves [1,73]. Most of these components of the feed ration are involved in the keratinization process, which ensures healthy horn growth and the structural binding of keratin proteins [1,74]. Horn keratinization is a complex and dynamic process that responds to stimuli (mechanical damage, trace element imbalance, and inflammation) in order to ensure the strength and integrity of the hoof [74,75]. Nutrients that are necessary for keratinization include amino acids (cysteine, methionine), fatty acids (linoleic and arachidonic acid), minerals (especially calcium), and trace elements (zinc) and vitamins (biotin) [73]. Imbalances in minerals (zinc, copper, selenium, and manganese) and vitamins (especially A, D, and biotin) as well as other nutritional deficiencies can lead to the growth of a fragile horn, which may be more prone to cracks and infections [76]. Zinc, sulphur, and some trace elements positively affect the hardness of the hoof horn [77].

## 4. Lesions Causing Lameness

Lesions that cause lameness in cows can be of infectious or non-infectious origin [15,16,28,78,79]. The prime infectious lesions causing lameness are digital dermatitis [15,16,37,78,80] and interdigital phlegmon [16]. Non-infectious lesions are associated with the loss of tissue integrity due to traumatic events, excessive wear, nutritional deficiencies, or unduly hoof modification, which may provide the primary gateway for infection. Non-infectious lesions include laminitis [9,15,16,60], which is associated with metabolic disease [9,12] and was reported to be one of the most common causes of lameness in dairy cattle [81]. Other lesions of limb disease include white line disease, sole ulcers [15,16,60], heels erosion, hemorrhages, and others [37,60]. Cows that have suffered from hoof disorders in the past are more likely to develop other diseases, especially white line disease, digital dermatitis, sole ulcers, and hemorrhages [9], which are related to feeding [9,17]. The risk of hoof infectious diseases has been reduced in animals that were fed a diet containing increased neutral detergent fiber [79]. At the beginning of lameness, cows reduce feeding time and frequency by 44–46%, but their daily feed intake is not affected. The reason is that they compensate for the shorter time spent feeding by increasing the rate of feed intake [82]. In severely lame cows, there is a significant reduction in the feed intake [83]. It is necessary to prevent sudden changes in the feed ration, which cause changes in the composition of the rumen microbiota, the onset of decarboxylation processes of protein cleavage, and higher production of volatile fatty acids and lactic acid [2]. The microbiota in the rumen is not quick enough to adapt to rapid changes in the feed rations, which can lead to disorders of the forestomach function [84,85]. 

## 5. Laminitis

Laminitis may appear as a primary condition, but it is more often secondary to rumen acidosis [9,10,15,42,81,86,87,88,89,90,91], in which the risk of lameness increases [26,66,71,92]. Fructans and glucose have been shown to increase lactic acid production, thereby increasing the risk of laminitis [93].

Laminitis can be caused by cows not being able to consume enough dry matter during the first stage of lactation to meet high production requirements [4,15,94]. Farmers often try to compensate for this deficiency by feeding more concentrated feed with easily digestible forms of carbohydrates, which increases the energy value of the feed [94,95]. The administration of concentrated feed increases the production costs and improves animal production, because it increases daily weight gain and shortens fattening time [87]. As carbohydrates increase, fiber, saliva production, and chewing time decrease in the feed, and consequently the rumen pH decreases [26,27,35,42,94] due to the higher concentration of lactic acid produced by bacteria [9,26,40].

The differences in the proportion of starch in the feed ration of cattle may promote rumen damage with systemic adverse effects on other tissues, including the digital cushion, which is associated with pathologies that occur in the joint and laminar tissue. These structural changes in the hoof can predispose the animal to lameness and the development of hoof injuries, leading to economic losses [12,87]. In cows with laminitis that were fed a highly concentrated feed ration, a lower layer of fat on the heel was observed [87].

Various buffers, rumen modifiers (monensin, lasalocid, tylosin, and virginiamycin), and increased intake of copper, zinc, and biotin can help to alleviate the severity of laminitis and delay its onset [35]. Preventive strategies include gluconeogenic precursors added to feed or the modulation of rumen fermentation to increase rumen propionate production. Ionophore antibiotics, especially monensin, are enhancers of propionate production. It has been widely used in many countries to prevent ketosis and related diseases. However, their prophylactic use was banned in Europe in 2006. In 2013, monensin was reintroduced on the European market as Kexxtone1 (Elanco, Indianapolis, IN, USA) in order to prevent hyperketonemia and associated diseases in high-risk animals [96]. The use of modifiers has not been sufficiently investigated to determine whether they affect the health of the hooves [93]. A buffer in the form of a sodium bicarbonate supplement in the feed ration alleviates the decrease in rumen pH, which is observed in rumen acidosis [42].

## 6. Sole Ulcers and Hemorrhages

Some studies suggest an association between clinical laminitis and sole ulcers. A higher forage ratio, more concentrated feed, and higher protein intake in the feed result in a higher locomotion score. The lesions that are associated with lameness include sole ulcers and hemorrhages [26,81,88]. Thus, hemorrhages of the sole may be a symptom of subclinical laminitis.

In another two-year study, during the experiment, when the animals were fed concentrated feed, the number of sole ulcers significantly increased [97]. A Swedish study determined an eminent correlation between sole ulcers with concentrated feeds fed less than four times a day, time spent feeding, and feeding carbohydrates earlier than forage [81]. In cows that were fed 6 kg of concentrate per day before parturition, a significant increase in sole hemorrhage was reported as compared to cows fed 1 kg of concentrate per day [26].

Bergsten et al. [81] conducted a study in which one group of cows was fed carbohydrate feed; the other group had the same amount of concentrates, but was given free access to forage. In the first group, 68% of cows showed signs of clinical laminitis during calving, followed by an ulcer two to three months later in 64%. In the second group, clinical laminitis occurred in 8% of ulcers. Further suggesting that hemorrhages may be a symptom of subclinical laminitis [81]. Similarly, in a study conducted by Peters et al. [98] in a similar experiment, feeding a more concentrated feed led to a significantly higher incidence of ulcers.

## 7. Immune System Reactions

When compared to roughage, the highly concentrated feed ratio increases the expressions of interleukin-1β, interleukin-6, tumor necrosis factor α, and MMP-2 mRNA in lamellar tissues. The change in the composition and function of bacteria in the rumen that is caused by concentrated feed leads to higher levels of lipopolysaccharide in the peripheral blood and it can further activate the inflammatory response in the lamellar tissue and even cause laminar damage [26,81,86,87]. Lipopolysaccharide is a significant contributor to damage and local inflammation in the capillaries of the lamellae in the hoof [86], thereby disrupting the production of quality horn [26].

## 8. Effect of Nutrition on Lameness and Laminitis

### 8.1. Carbohydrates

In the current intensive production system, the feeding for cows includes a highly concentrated feed ration. Sudden changes in nutrition, especially an increase in easily hydrolysable carbohydrates and/or a decrease in the quality and amount of fiber in the feed, is usually the main cause of laminitis [6,9,15,19,26,35,36,40,81,92,93,95,99,100] and it is associated with dermatitis digitalis [15]. Farms on which heifers were fed a ration of maize silage had a higher locomotion score than those on which they were fed a ration without a proportion of maize silage [81,92,101]. Dairy cows that are fed maize silage for a long-time experience limb disease (laminitis, interdigital dermatitis) [102]. The study by Amory et al. [101] reports ten farms on which heifers were fed maize silage as part of their feed ration had a higher locomotion score than those on which no maize was fed. A possible hypothesis for this association is that maize silage causes metabolic disturbances due to rapid rumen fermentation and subsequent acidosis [15,86,103]. These metabolic disorders reduce the quality of horn formation [73] and they may predispose a cow to lameness due to the lower resistance to hoof horn abrasion. The integrity of the rumen epithelium also appears to be important for controlling the relationship between acidosis and laminitis due to its potential for toxin absorption [87]. The high proportion of silage in the feed ration significantly reduced the hardness of the abaxial wall of the hoof and sole and increased the growth of the hoof horn, which increased the incidence of lameness [92]. The reduced hardness of the hooves when fed with a high ratio of concentrated feed was also reported by Burger [26].

Not only the composition of the diet, but also the method of preparation, feeding, and feeding behavior of animals, are important risk factors for the development of laminitis [81]. In contrast, Coombe et al. [88] did not find a significantly higher incidence of white line disease, ulcers, or hemorrhages in dairy cows on pastures and in stables in different stages of lactation when fed silages. Ranjbar et al. [6], who fed 3 to 14 kg of grain per cow per day and did not notice a significant difference in the amount of carbohydrates fed and the incidence of lesions, obtained the same result.

### 8.2. Fats

Associations between the fat layer of the heel and the nutritional status [9,93] suggest that dietary lipid precursors, including lipids that formed from feed and those derived from short-chain fats, may affect lameness [93]. The digital cushion may be more vulnerable if the cows are fed a low-fat feed ration or if antioxidants, such as vitamin E, have a lower concentration. Diets containing temperate grasses, such as ryegrass (*Lolium* spp.) and fodder and grain, have lower lipid content, which may pose a risk to hoof integrity. The occurrence of ulcers depends on the height of the digital cushion. Skinny cows mainly due to the negative energy balance after calving have a smaller digital cushion, so they are up to nine times more susceptible to hoof diseases [93].

Anti-inflammatory n-3 polyunsaturated fatty acids may play a role in the development of laminitis and dermatitis digitalis [66]. Maize usually has a high proportion of linoleic acid. Linoleic acid is the basic n-6-polyunsaturated fatty acids, which is a precursor of endogenously metabolized arachidonic acid. Roughage, on the other hand, usually contains more linolenic acid, the basic n-3-polyunsaturated fatty acids. Linolenic acid is a precursor of eicosapentaenoic acid, which reduces the production of arachidonic acid and it is also a precursor of eicosanoids with anti-inflammatory and immunosuppressive effects, such as reduced lymphocyte proliferation or natural killer cell activity. The composition of fatty acids in the digital cushion, as in other tissue lipids, can be affected by diet [104].

### 8.3. Proteins and Amino Acids

Dietary risk factors that are associated with lameness and laminitis include clinical and subclinical rumen acidosis [6] and high protein levels, along with low fiber concentrations [6,26,92]. A higher protein content in the feed ration (19.8%) significantly increased the locomotion score, number and duration of lameness in dairy cows between three and 26 weeks after birth than feed with lower protein content (16.1%) [26]. High levels of rumen-degradable proteins increase the risk of lameness and laminitis [26,35]. Excess protein in the feed ration can be problematic due to faster horn growth [105]. Ammonia has a toxic effect, and high concentrations of ammonia or urea in the blood can damage sensitive lamellae and corium in the hoof [93]. Rumen ammoniacal nitrogen is a nutrient that supports effective rumen fermentation. Its level is associated with the rate of protein degradation in the rumen, rumen degradable protein concentration and the amount of available energy in the diet for rumen microbiota. The extent to which ammonia is used to synthesize microbial protein is largely dependent on the availability of energy that is generated by fermentation carbohydrates [106]. Acidosis is reflected in an increase of volatile fatty acid production and decrease in the concentration of ammonia concentration and rumen pH. High rumen levels of ammonia can buffer changes in rumen pH [93].

#### Cysteine a Methionine

The amino acids cysteine and methionine, which contain sulphur, are essential for promoting the structural and functional integrity of the hoof, as they are involved in the formation of a disulphide bond during keratinization [19,66,73,93,105]. Microbial protein is an essential source of methionine, which is the basis for the construction of the hoof horn [2,107].

An inappropriate ratio of cysteine to methionine induces the production of a softer horn [4,35,73]. In high-yielding dairy cows, the production of these amino acids in the rumen is not enough to meet the high lactation requirements, even though sulphur is provided in the feed ration. In these cases, it is necessary to optimize the protein fraction in the feed ration [105]. The nutrition of high-yielding dairy cows is often based on increasing the amount of silage in the feed ration, and maize silage and grain have a low sulphur content; hence, the doses of sulphur provided may be insufficient [108].

A methionine feed supplement that was designed to improve the supply of sulphur amino acids has limited effects on the properties of the horn [66]. This finding contradicts those of Clark and Rakes [108], who fed a hydroxy analogue of methionine and observed faster hoof growth than the control group. The hooves of cows fed a hydroxy analogue of methionine contained less cysteine and proline than control cows and a higher percentage of methionine, lysine, tyrosine, and glutamic acid. These results suggest that there is a reduction in disulphide bonding in the hoof tissue of cows that were fed a hydroxy analogue of methionine; therefore, feeding with this supplement is not recommended [108].

### 8.4. Endotoxins and Histamine

Many studies have identified the potential of vasoactive substances, including histamine, tyramine, lactic acid, serotonin, and endotoxins, which are produced in the gastrointestinal tract and in systemic diseases, in order to affect the hoof blood capillaries network [93]. As the pH in the rumen decreases during metabolic acidosis, histamine, and other endotoxins are released into the blood, which can cause vasodilation, ultimately damaging the network of blood vessels in the hoof [9,26,35,40,66,94,105]. Histamine causes vasoconstriction, dilatation, laminar destruction, deterioration of hoof quality, and the process of laminitis develops [26,91].

When the feed ration is changed, the microbiota in the rumen also changes where lactic acid producing bacteria multiply and rumen pH decreases due to the different digestion of carbohydrates [2,26,93]. In this case, histamine is formed and the original rumen microbiota die [2,26,35]. The same negative effect occurs when feeding low-quality feed, especially rotten silage and moldy feed, which contain endotoxins [2,26]. Endotoxins are lipopolysaccharides that are released from the cell walls of Gram-negative bacteria [93] and stimulate histamine release [26,35,40,77,81], which causes vasoconstriction, resulting in an insufficient blood supply to the hoof dermis and, subsequently, an insufficient supply of oxygen and amino acids that are necessary for horn formation to the tissue [77]. These substances also slow the regeneration of the rumen epithelium [93] and activate enzymes that cause the degradation of the collagen fibers of the suspension apparatus, leading to the displacement and collapse of the distal phalanx [40,64].

### 8.5. Minerals

Dairy cattle are estimated to require at least 22 minerals in the feed ration [109,110], 17 minerals [110]. Table 2 lists the mineral requirements for dairy cattle [111].

Mineral and vitamin supplementation is associated with a reduction in the incidence of lameness in dairy cows. Improper mineral nutrition can also be a prerequisite for lameness [26,71,112], being mainly caused by a lack of calcium, phosphorus, zinc, copper, manganese, selenium, iodine, or sulphur amino acids [83]. Minerals play a considerable role in determining the structural strength of hooves due to their involvement in specific biochemical pathways that are associated with keratin synthesis [1,74]. Overall, methionine, cysteine, fatty acids, vitamins, and macro- and micro-elements are associated with lower hoof horn quality and susceptibility to lameness that is associated with the sole [105]. Minerals, calcium (Ca), iron (Fe), copper (Cu), zinc (Zn), iodine (I), selenium (Se), molybdenum (Mo), and chromium (Cr) affect the development and hoof disease in dairy cows [10]. The availability of trace minerals is influenced by factors, such as iron (Fe), sulfur (S), calcium (Ca), and toxic metal levels in the feed ration [113]. Appropriate mineral supplements can improve the immune response and hoof health of cattle [114,115].

#### 8.5.1. Trace Elements

Significant trace elements, such as zinc (Zn), manganese (Mn), and copper (Cu), are essential cofactors for a large number of enzymes [26,27,42,75], including those that are involved in keratin production, which are therefore associated with hoof health [26,42,74,75] and play a substantial role in maintaining redox balance and healthy development of the horn [27].

The low dry matter content in the early postpartum period disrupts the supply of most nutrients, including trace elements, due to the high demands on milk production. The supply of trace minerals (Zn, Mn, Cu, and Co) in more bioavailable forms, such as amino acid complexes (AACs), could help to reduce the incidence of hoof disorders in the peripartum period of dairy cows [75]. The use of trace elements from organic sources (complexed chelated amino acids, proteins) can be nutritionally important from a nutritional point of view to maximize milk production and maintain health [79]. The incidence of hoof heel erosion was lower in the group of cows that were given minerals in an amino acid complex. The prevalence of sole ulcers did not differ between the experimental and control groups. Dairy cows that were fed a feed ration with AACs reduced the need for regeneration of the hoof dermis cell pathways and increased the availability of biotin in the sole. These molecular changes may have been due to a lower incidence of heel erosion in response to the addition of AACs to the feed. The supplementation of the feed ration with AACs during the peripartum period affected the transcription of various genes that affect the structure, oxidative stress, and hoof inflammatory processes. The results of the study showed that AACs have positive effects at the molecular level on the horn of the sole [75].

Döepfer et al. [116] compared the effectiveness of a new nutritional supplement containing trace elements (zinc (Zn), manganese (Mn), and copper (Cu)) in the form of amino acid complexes and cobalt glucoheptonate with a supplement containing trace elements, predominantly inorganic in nature, on the occurrence and course of digital dermatitis (DD) in heifers. In their study, they found that supplementation in the form of amino acid complexes reduced the overall rate of increase in the prevalence of dermatitis digitalis lesions (active M2 + chronic M4 lesions) when compared to supplementation in inorganic form [116].

Similarly, the incidence of white line disease, hemorrhages, sole ulcers, and double sole was reduced when cows were fed other Zn, Mn, Cu, and Co supplements in a complex form. Organically-bound zinc and cobalt, after the addition of manganese and copper complexes, improve the integrity of the horn [113]. Dairy cows that were fed mixtures supplemented with chelated zinc improved the integrity and hardness of the hoof horn [115,117]. Trace elements that were bound in organic form administered 75 days after parturition reduce the incidence of white line disease and heel erosion; 250 days after farrowing, cows had a lower prevalence of white line disease, foot rot, and sole ulcers [113]. If cows developed a lesion, supplementation with trace elements in organic form reduced its severity. However, these cows fed trace elements bound in organic form had a higher incidence of hemorrhages (only 4%) than dairy cows fed inorganic trace elements [113]. The lower incidence of lesions is associated with an increase in the hardness of the horn after supplementation of the feed ration with trace elements. Harder horn has been shown to contain higher concentrations of zinc when compared to softer-heel horns, and lame animals also have significantly lower Zn concentrations in the horn as compared to healthy animals [117]. Lower contents of zinc [19,27], copper, manganese, and calcium were also detected in the serum and hair of lame dairy cows [27], as well as iodine, iron, and selenium [10]. Significantly increased concentrations of chromium (Cr) were also detected in lame dairy cows, and the levels of molybdenum (Mo) showed a decreasing trend [10].

Silage can provide up to 9% Ca, 34% K, 23% Mg, 12% P, and 15% S of respective daily needs for dairy cattle consuming 26 kg dry matter per day composed of 40% (10.4 kg DM) corn silage. For trace elements, the average corn silage can provide up to 13% Cu, 847% Fe, 80% Mn, and 11% Zn from daily intake requirements [110].

##### Zinc

Zinc is crucial for hoof formation, providing catalytic, structural, and regulatory functions in keratinization [4,27], playing a considerable role in the creation of structural keratin proteins [27,42,73,103,115] and regulating calmodulin and responsible for the transfer of calcium ions to the cytosol of keratinocytes, which is relevant in the last stage of keratinocyte development [19,93]. Zinc is significant for maintaining the health and integrity of the skin due to its role in cell repair and replacement and it plays a crucial role in wound healing [65,73,103,114,115,118]. Feeding a balanced mineral mixture with the addition of zinc is suitable for preventing interdigital necrotic inflammation [84]. Zinc deficiency is also often involved in the development of inflammatory processes on the skin and distal limb (digital dermatitis) [2], parakeratosis and hyperkeratosis in the hoof epidermis, and nutrition and horn production are interrupted, so disorders of hoof growth [117] and subsequent lameness of cows occur [117,119]. The results of a study conducted by Zhao et al. [27] showed a reduced amount of Zn, Cu, and Mn in the horn in lame cows, which may have been induced by the insufficient deposition of Zn, Cu, and Mn in the body.

Zinc concentrations in cows with chronic laminitis are significantly lower than in healthy cows [89]. Zinc concentrations, in a study by Belge et al. [89], ranged between 8.46 and 44.92 μmol/L (mean 20.54 ± 1.19 μmol/L) in cows with chronic laminitis, and between 19.05 and 30.77 μmol/L (mean 24.66 ± 0.87 μmol/L) in healthy cows. [89].

Negative interactions between trace elements and macroelements and dietary factors pre- and post-absorption may further reduce their bioavailability [95,120]. Supplemented zinc for dairy cows is usually in inorganic form (zinc oxide or zinc sulphate). Recently, a new source of elemental Zn has been used in animal nutrition. The development of nanotechnologies has led to the creation of Zn nanoparticles, which have unique properties, including a large specific surface area, high surface activity, high catalytic efficiency, and strong absorption capacity. Zinc nanoparticles (ZnN) are specially prepared mineral salts with a particle size from 1 to 100 nm. ZnN feeding has been shown to have higher efficacy and reduced toxicity as compared to conventional Zn sources [115].

Although most studies recommend supplying minerals in organic form, Noori et al. [42] successfully used zinc in the form of sulphate for fattening bulls for the decrease the occurrence of lameness. Singh et al. [19] successfully used zinc together with biotin in the form of sulphate in their study in order to prevent the occurrence of hoof diseases. The study conducted by Faulkner et al. [114] reported a possible positive effect that organic zinc may have on the incidence of dermatitis digitalis in cattle. Faulkner et al. [114] found that feeding organic forms of zinc contributes, amongst other things, to reductions in the colonization of bacteria (*Treponema* spp.), which cause digital dermatitis. Thus, this study showed another possible positive effect of organic zinc on the incidence of dermatitis digitalis in cattle [114].

Zinc and biotin are often combined in studies. The feed ration with biotin and zinc significantly increased plasma biotin concentrations and Zn levels in the supplemental groups as compared to the control. By feeding supplemental nutrients (biotin and zinc), lameness can be minimized in high-yielding dairy cows during the parturition period [118].

##### Copper

Copper (Cu) is part of protein synthesis, vitamin metabolism [113], connective tissue formation, and the immune system [42,65,113,120,121]. Copper is an essential element for the activation of the thiol oxidase enzyme, which is responsible for the formation of chemical bonds between keratin fibers. This process is necessary for the structural strength of the cell, providing the rigidity of the keratinized cell matrix. Cattle that suffer from subclinical copper deficiency are more susceptible to hoof diseases [27], such as heel cracks, footrot, and sole ulcers. Cell-mediated and humoral immunity are both significantly suppressed by copper deficiency [65].

Copper plays an extensive role in the maturation of keratin and the formation of connective tissue [42,93]. Copper can be supplemented in inorganic copper sulfate or copper methionate. Excess molybdenum increases copper needs [93]. Insufficient amounts of zinc, copper, and manganese promoted oxidative stress, increased incidence of cartilage disorders, and poor horn quality [27]. Copper bound in an organic amino acid complex is more bioavailable than inorganic sulphate sources [103,112,113,114,115,118,121], thereby improving the production of keratinized tissue [113,117], demonstrating a lower incidence of hoof disease in dairy cows fed organic forms of trace elements [113] and a reduction in the prevalence of lameness [27].

##### Manganese

Manganese (Mn) plays role in protein synthesis, vitamin metabolism [113], connective tissue formation, the immune system [18,65,113,120,121], and the rate of wound healing [65,121]. A lack of manganese causes poor hoof quality, which reduces their hardness, fragmentation, and disorganization of collagen fibers with reduced collagen content [27,93]. Manganese plays an indirect role in the keratinization process and is essential for the activation of the enzymes galactotransferase and glycosyltransferase, which are required for the synthesis of the side chains of chondroitin sulphate proteoglycan molecules [27,93]. Proteoglycans are important building blocks in the formation of healthy cartilage and bone [27]. Manganese absorption is low in cattle and it is adversely affected by higher levels of calcium, phosphorus, and iron in the feed ration. Manganese is supplemented as chloride, sulphate, carbonate, or manganese oxide.

##### Selenium

A positive relationship of selenium (Se) to hoof tensile strength was noted. Selenium plays a role in protecting the structure of the horn from oxidative damage due to the binding of keratin proteins [1,93]. None of the other minerals mentioned have a significant effect on the tensile strength, including zinc, which has been shown to play a notable role in the structural strength of hooves [1]. Selenium protects and maintains the intercellular mass of keratinocytes, which is rich in lipids [4,93,105].

Selenium acts together with vitamin E. Selenium from an inorganic form is passively absorbed in the intestine and it is immediately used for the synthesis of some selenoproteins. Selenium in the organic form of *Saccharomyces cerevisiae* or selenomethionine (selenocysteine) is much more expensive. Selenium in organic form is better absorbed and stored in tissues, but the differences in retention are not very large [93]. Selenium levels are low in many pastures, and supplementation is necessary to increase Se intake and maintain the status of Se grazing dairy cows. Herds with medium concentrations of Se in the blood have higher milk production and better reproductive performance.

##### Iodine

The addition of a noticeable amount of iodine (I) to the feed ration produces a potential improvement in hoof health. However, iodine supplementation can lead to excessive amounts of iodine in milk, so this supplementation is especially suitable for fattening bulls. The solution may be a rapeseed meal, which contains goitrogenic compounds that reduce the transfer of iodine to milk. Higher iodine content in the feed ration leads to a reduction in the incidence of digital dermatitis, and hoof lesions are smaller [122]. Iodine affects the local inflammatory response and the ability of macrophages to form compounds that destroy pathogens [65,121].

In beef cattle, the effects of feed with inorganic and organic forms of trace minerals were compared while using feed with similar concentrations of trace elements exclusively from inorganic sources on prevalence of and impact on digital dermatitis. The study aimed to elicit a better immune response of the animal to digital dermatitis and improve the integrity of the epidermis and resistance of the sole, together with more frequent inspections and the use of preventive disinfection baths. Initial serum concentrations of total iodine were not significantly different between the groups. After 60 and 90 days, the feed intake groups with increased addition of trace minerals in organic form and iodine showed better results in controlling dermatitis digitalis and reducing active M2 and M4 lesions [121]. In contrast, in another study, a group of cattle that were fed an iodine premix had higher serum iodine levels than the control group; however, feeding trace elements bound in organic form with the addition of iodine achieved a reduced size and number of M2 lesions [65].

#### 8.5.2. Macroelements

##### Calcium

Calcium plays a significant role in keratinization and it is essential for the final stage of mature horn cell formation [27,31,73]. Insufficient calcium during keratinocyte maturation can lead to a dyskeratotic horn [27]. Calcium (Ca) concentration has a negative relationship to the tensile strength of the horn. Calcium plays a role in keratinocyte formation by activating the enzyme epidermal transglutaminase, which is involved in cell membrane formation [1,19,31,73]. Higher calcium concentrations in the hooves of dairy cows increase their hardness [1].

There is some speculation that normocalcemia and a mild deficiency of calcium when associated with lower magnesium concentrations may be related to circulatory failure in the digital tissue of lame cows [31]. Casagrande (2013) reported that hypocalcemic animals show higher hoof wall than normocalcemic animals [123]. A study by Barbosa et al. [31] reported that serum calcium levels were lower in lame cows when compared to healthy cows. Lame cows showed concentrations of calcium, indicating subclinical hypocalcemia.

##### Phosphorus and Magnesium

Phosphorus (P) and magnesium (Mg) are also an integral part of building healthy hoof and bone. Phosphorus and calcium are bound by a specific ratio for proper hoof growth, whereas poor ratios can cause hoof horn fragility. Magnesium helps the distribution and absorption of calcium, and its deficiency can directly lead to insufficient production in bone formation and affect their strength [27,93]. Manganese plays an indirect role in keratinization, mainly as an activator of pyruvate carboxylase. This enzyme is involved in energy production [93].

Magnesium (Mg^2+^) is an essential mineral with many cellular functions [123,124]. The primary site of Mg^2+^ absorption is the rumen [123] and may be influenced diet type and forage type [125]. The ruminal transport of magnesium is significantly affected by several factors, such as high potassium concentrations, sudden increases in ammonia, pH, and short-chain fatty acid concentrations [123].

In a study conducted by Jelinski et al. [126], lame cattle had significantly lower concentrations of magnesium in the hoof wall and sole compared to the control. With each 10ppm reduction in magnesium, the probability of diagnosing tip necrosis syndrome increased by 1.13-fold at the hoof wall and 1.21-fold at the sole [126].

### 8.6. Vitamins

#### 8.6.1. Vitamins A, C, D, and E

Vitamins A, D, and E play a notable role in the structure and quality of the hoof horn [26,93,127]. Vitamin A is involved in the differentiation of keratinized cells [93,105] and it affects epidermal transglutaminase [73]. Vitamin D regulates calcium metabolism and affects keratinization. Vitamin E is eminent for maintaining the cell membranes and intercellular mass of the hoof horn [93]. Linoleic and arachidonic acids and biotin (a coenzyme of linoleate and arachidonate metabolism) are produced by the rumen microbiota and they are limiting for hoof integrity as they form a barrier against the loss of water from the horn and increase the consistency and resistance of the hoof. Vitamins A, C, and E protect the above fatty acids from oxidation. A lack of these amino acids, fatty acids, and vitamins is associated with lower hoof horn quality and a predisposition to lameness that is associated with the sole [105].

#### 8.6.2. Biotin

Biotin, vitamin B7, is a significant vitamin for keratinization [19,30,61,73,75,81] and hoof integrity [26,75,81,93,118,128], which increases the importance of biotinidase, the enzyme responsible for recovery and recycling biotin [75]. This water-soluble vitamin [18,61,81,129], which is also called vitamin H [19,73,81,129,130], acts as a cofactor in gluconeogenesis, fatty acid, and protein synthesis [18,61,73,81,96,128]. Biotin is a cofactor of microbial enzymes that is involved in the synthesis of propionic acid [128]. Biotin is an essential factor for the production of several carboxylase enzymes that catalyse substantial steps in intermediate metabolism [41,66,72,73,81,107,129]. The role of biotin in lipogenesis, as a cofactor in acetyl coenzyme A (CoA) carboxylase and the synthesis of higher fatty acids, affects the amount and composition of fatty acids in the heel cushion [18,73,81,129].

Biotin is essential for the production of lipids in the intercellular mass and, together with zinc and copper, enables the growth of resistant horns [93,119]. Biotin affects the proliferation and differentiation of the epidermis; it is also essential for normal keratinization [18,30,67,72,81,107]. Biotin is associated with a reduction in the incidence of lameness [30,93,109], specifically reducing the prevalence of white line disease [18,30,41,67] by improving its structure and strength [30,107] by 45% [60]. Sole hemorrhages were significantly higher in the control group (50%) as compared to cows to which biotin was added to the feed ration (24%) [81]. The incidences of double sole, horn cracks, and heel erosion were the same between the control group and biotin-supplemented cows [41,81], which is not consistent with the results reported by Randhaw et al. [18], in which they noted a significant decrease in the incidence of the heel erosion and double sole after the addition of biotin. The biotin concentrations are significantly lower in lame cows [41,42,61,67,72,81]. The administration of 20 mg biotin per day to dairy cows significantly reduced the incidence of lameness [30,41,107,131]. Biotin supplementation increased the sulphur content and decreased the levels of calcium and potassium in the abaxial hoof wall [131].

Biotin has been found to increase the rate of lesion healing in cows and it has a positive effect on the structure and quality of the new horn [18,30,67,94,131] during the ulcer healing process [30]. Feed supplementation with biotin improved the macroscopic appearance of the hooves [61,67]. On the contrary, the study by Queiroz et al. (2020) reported that biotin treatment did not affect the surface relief of the hoof wall, suggesting that its action is limited to the inner layers of the stratum corneum [131]. The results of histological and biochemical studies indicated that the inter- and intracellular ultrastructures of the horn were improved [30,61,81,131]. In the absence of biotin, the horn had a whitish color and brittle and friable consistency [73]. Biotin supplementation created a more defined and cohesive structure [30,107] and the horn was harder [107], so it is recommended to include biotin in the feed ration as a precaution [30,61,128]. Higuchi and Nagahata [61] found a significant increase in moisture in the horn of the sole of biotin supplemented cows, in contrast to the control, but Chen et al. [107] claimed that the humidity was not increased. The water content is associated with the microscopic and biochemical structure of the hoof horn. This study suggested that changes in the hoof horn that are caused by laminitis result in the production of a softer horn with higher moisture, which is more prone to damage, and a reduction in available biotin may result in impaired keratinization and consequent horn quality. Changes in the horn structure during laminitis are reminiscent of horn changes in calves suffering from biotin deficiency [61]. The lack of biotin harms the quality of the horn and conditions the formation of cracks in the hoof capsule [4]. High doses of biotin can reduce these horn cracks [61].

Feed rations generally contain significant amounts of B vitamins and rumen bacteria can also synthesize large amounts of B vitamins [132]. Microorganisms in the rumen synthesize biotin [19,30,107,128,130], in varying amounts depending on the composition of the diet [81], and other B vitamins; therefore, an absolute deficiency of biotin has not been demonstrated in ruminants [30,67,107]. However, in high-yielding dairy cows that were fed a diet high in carbohydrates, the synthesis of biotin by the rumen microbiota is insufficient. Therefore, it is necessary to add biotin to the feed [107,128]. However, there is evidence that rumen acidosis [30,42,61] or feeding poor quality feeds [2] may reduce biotin synthesis [2,26,30,61] and reduce the production of microbial protein [2,30,42,61].

### 8.7. Mycotoxins, Poor Feed Quality and Toxicity

The health of the limbs is also affected by dietary disorders that are caused by an improper feed ration or poor feed ration structure. Moldy feeds can significantly affect the development of hoof inflammation [83]. Intake of moldy feed is manifested by yellow or red discoloration of the horn of the sole, which is caused by hematomas [35,133]. If care is neglected, this can lead to sole ulcers [83]. Jensen et al. [70] reported that the microbiological and sensory quality of silage is often not good, even in control farms. Systemic metabolic diseases, feeding with mycotoxins, and other nutritional deficiencies cause rumen acidosis, which often leads to laminitis in cattle [35,62,105]. Aflatoxins, which are one of the most significant mycotoxins, cause reduced growth and reproduction, increased susceptibility to injury, reduced feed intake, the disruption of the cellular and humoral immune system, and cause subclinical laminitis [100]. The moist feed ration contributes to an increase in the incidence of heel erosion and, thus, leads to a higher prevalence of hoof disorders [9].

There is a potential relationship between nitrate toxicity and laminitis [93]. Plants with a high nitrate composition can cause subsequent poisoning of grazing animals. Nitrate tastes bitter, which reduces the palatability of nitrates diet and may cause lower feed intake [134]. Nitrate is converted to nitrite in the rumen [93,135], which converts haemoglobin to methaemoglobin, thus reducing the blood’s ability to carry oxygen [93,134]. Nitrites are also a potent vasodilator. Stagnation and association blood in the peripheral circulation, including the vascular capillaries of the corium, can cause anoxia and accumulation of toxins in tissues, potentially increasing the susceptibility to laminitis [93]. Gontijo et al. [135] report poisoning in cattle by nitrate/nitrite after the ingestion of creeping rivergrass (*Echinochloa polystachya*), elephant grass (*Pennisetum purpureum*), oat (*Avena sativa*) and ryegrass (*Lolium* spp.). Other forage species with high nitrate content are maize (*Zea mays*) and sorghum (*Sorghum* spp.) [135].

Fungi infecting grasses or grains produce toxic alkaloids (ergot alkaloids, ergovalin, Loliter B), which are absorbed into the bloodstream after ingestion by animals and have a toxic effect on the organism. Symptoms of poisoning occur mainly in grazing animals [136]. Ergotic poisoning is the result of ingestion of alkaloids [93], which are mainly associated with ergotamine [137], produced by the fungus *Claviceps purpurea* [93,137] usually found on wheat, barely, and perennial rye and tall fescue and cereals [137]. This poisoning can result in lameness, swelling, and gangrene of sole and distal part of the hindlimb, and loss of the limbs due to vasoconstriction [93,137].

### 8.8. Pasture

One of the factors that could have a significant effect on lameness is the use of pasture [24]. Pasture-breeding cattle usually have a lower incidence of lameness than stable-breeding cattle [7,20,36,68], but grazing cows still experience limb problems, as evidenced by reports of a lameness prevalence of up to 76% [7] and in Wisconsin from 0% to 54% (average 8%) [58]. The study conducted by O’Connor et al. [20] reports a prevalence of 85% of non-infectious diseases and 2.8% of infectious diseases in grazing cattle, which is related to environmental hygiene, which is worse in stables, where the ratio of infectious and non-infectious diseases is usually opposite. Grazing can have a protective effect on cattle limbs and it can reduce the risk of specific lesions or diseases (sole ulcers); yet some grazing systems can potentially increase lameness [23]. Therefore, the rearing of cows in pastures seems to improve welfare only temporarily in terms of lameness compared to cows kept in stables [46].

High-yielding dairy cows may not be able to satisfy their nutritional requirements exclusively from grazing and, therefore, lose weight and milk production decreases. Some hoof disorders have a higher prevalence in zero dairy grazing. The beneficial effects of seasonal grazing on lesions, such as hemorrhages, sole ulcers, and heel erosion, cannot be verified. Grazing systems have a preventive effect on infectious diseases of the hooves (dermatitis digitalis and interdigitalis, erosion of the heel), but this is due to a lower probability of traumatic lesions and the absence of direct contact of infectious lesions with contaminated manure. Dermatitis digitalis is surprisingly the most common lesion reported in many studies in grazing cattle in Brazil [46,78].

The lower nutrient content of grasses compared to total mixed ration (TMR) leads to a change in the time spent feeding, feed intake, rest, and ultimately to a longer period of feed intake in grazing cows [46]. Grazing cows can develop subacute rumen acidosis due to a combination of high-quality pastures [35,46] with high water content and high concentrations of rumen-degradable protein [35], irregularities in dry matter intake [46], or high carbohydrate feed intake [35,46]. These pastures can then increase the risk of laminitis, especially if they are supplemented with kernels or other feeds that contain significant amounts of starch [35].

### 8.9. Yeast

Cultures of *Saccharomyces cerevisiae* are often added to the ration for dairy cows in order to improve rumen fermentation and promote changes in rumen digestive processes. It can also be added to the feed ration at the beginning of lactation to improve the health of the cows and their nutritional status. Yeast cultures stabilize rumen pH [29,138] by stimulating the growth of rumen bacteria, which use lactic acid that can minimize the risk of rumen acidosis and, thus, predisposition to laminitis and subsequent lameness [29,138]. Yeast cultures in the feed ration do not affect the prevalence of lameness in cows, but have the effect of reducing the locomotion score [29]. Yeast culture, especially Saccharomyces cerevisiae, often improves digestibility of neutral detergent fiber [138,139] could increase acetate, but also increase the production of dry matter intake and milk. Yeast increases the availability of dicarboxylic acids, which perform central metabolic reactions and serve as electron carriers [138].

## 9. Oxidative Stress

Oxidative stress plays a significant role in the lameness of dairy cows caused by disorders of mineral metabolism [27,93]. Oxidative stress contributes to the pathogenesis of lameness by causing an imbalance in selective minerals that promote healthy hoof growth. Trace elements are known to be important for reducing free radicals at the cellular level and affecting the balance of antioxidants and free radicals [27]. Analysis of the plasma mineral profile in lame dairy cows revealed a significant decrease of concentrations copper, calcium, and phosphorus levels as compared to the control. The antioxidant system is disrupted, and such animals need the addition of minerals, especially calcium, phosphorus, and copper [140].

Al-Qudah and Ismail [72] provided evidence for the potential role of oxidative damage in the pathogenesis of lameness. A strong association has been found between serum biotin and antioxidants and oxidation profiles in cows with hoof disorders [72].

## 10. Prevention

Excessive feeding of cows during the dry period causes hyperinsulinemia and hyperglycemia in the early phase of lactation, which are classic symptoms of insulin resistance. It is important to ensure that dairy cattle are provided high quality roughages to reduce the incidence of laminitis [4]. During the dry period, dairy cows should be fed a small dose of concentrates, or none. The dose should be increased just before and after calving. It is relevant to ensure enough good quality roughage is fed [84]. A high proportion of concentrates in feed rations, which contain more than 28% starch, has undesirable effects on the usefulness and health status of animals, including hoof diseases. Emphasis is placed on maintaining the adequate physical condition of the animals and preventing them from becoming overweight [141]. It is imperative that changes to the composition of feed rations are made gradually, and that dairy cows are provided enough roughage for proper ruminating to minimize the occurrence of laminitis [77,132].

## 11. Conclusions

Nutrition is one of the most relevant risk factors for cattle lameness, which is currently a critical problem in dairy farming, affecting animal health and welfare. It is a crucial preventive factor, as it affects the growth and quality of the horn. Horn growth is a complex process that requires cysteine, methionine, minerals, especially calcium, zinc, and vitamins, especially biotin, for its keratinization. Nutritional deficiencies lead to the formation of a fragile horn, which is prone to cracks and infections. Sulphur and zinc have a positive effect on the hardness of the hoof horn. Non-infectious diseases that are associated with metabolic disorders include, in particular, laminitis. In lame cows, feed intake decreases by 44–46%. Breeders want to compensate for this decline by feeding concentrated carbohydrate feeds, and it leads to rumen acidosis and subsequently laminitis. A similar toxic effect can cause high levels of ammonia or urea in the blood. Rumen acidosis is a predisposition to lameness in dairy cows, due to the higher abrasiveness of the horn. In the case of diseases of limbs, prevention is necessary, which is economically much less expensive than the treatment of painful lesions. The importance of limb health in dairy cows is expected to increase with the increasing intensification of livestock farming.

## Figures and Tables

**Table 1:** Prevalence of lameness in dairy cows in different states.

State	Prevalence (%)	Reference
America		
North America	2–55	[11]
	55	[44]
Minnesota (USA)	24.6 (3.3–57.3)	[25]
Canada	20–35	[45]
Brazil	35	[23]
Australia and New Zealand		
Australia	7.5	[35]
	18.9	[6]
New Zealand	14	[35]
Africa		
Kenya	23	[46]
	11.7	[47]
Algeria	12.7	[48]
Europe		
England and Wales	36.8 (0–79.2)	[49]
France	25 (0–51)	[25]
Germany	20 (0–79)	[25]
Austria and Germany	34	[50]
Sweden	5 (0–25)	[25]
	14.2	[51]
Spain	10 (0–27)	[25]
	13.8	[52]
Switzerland	14.8	[53]
Asia		
Malaysia	19.1 (10.0–33.3)	[13]
India	17.2	[54]
	8.1–30.5	[55]
Thailand	21.98	[56]
China	31	[57]

**Table 2:** Mineral Requirements for dairy cattle.

Minerals	Growing and Finishing Cattle	Gestating	Early Lactation
Zinc, mg/kg	30	30	30
Copper, mg/kg	10	10	10
Manganese, mg/kg	20	40	40
Selenium, mg/kg	0.10	0.10	0.10
Iodine, mg/kg	0.50	0.50	0.50
Magnesium, %	0.10	0.12	0.20
Sulphur, %	0.15	0.15	0.15
